# Transbronchial needle aspiration “by the books”

**DOI:** 10.4103/1817-1737.78427

**Published:** 2011

**Authors:** Elif Kupeli, Leyla Memis, Tugce S. Ozdemirel, Gaye Ulubay, Sule Akcay, Fusun O Eyuboglu

**Affiliations:** *Pulmonary Diseases Department, Baskent University School of Medicine, Ankara, Turkey*; 1 *Department of Pathology, Gazi University School of Medicine, Ankara, Turkey*

**Keywords:** Bronchoscopy, lung cancer, mediastinal lymphadenopathy, sarcoidosis, transbronchial-needle aspiration

## Abstract

**BACKGROUND:**

Training for advanced bronchoscopic procedures is acquired during the interventional pulmonology (IP) Fellowship. Unfortunately a number of such programs are small, limiting dissemination of formal training.

**OBJECTIVE:**

We studied success of conventional transbronchial needle aspiration (C-TBNA) in the hands of physicians without formal IP training.

**METHODS:**

A technique of C-TBNA was learned solely from the literature, videos and practicing on inanimate models at “Hands-On” courses. Conventional TBNA with 21 and/or 19 gauge Smooth Shot Needles (Olympus^®^, Japan) was performed on consecutive patients with undiagnosed mediastinal lymphadenopathy.

**RESULTS:**

Thirty-four patients (male 23), mean age 54.9 ± 11.8 years underwent C-TBNA. Twenty-two patients had nodes larger than 20 mms. Suspected diagnoses were malignancy in 20 and nonmalignant conditions in 14. Final diagnoses were malignancy 17, sarcoidosis 4, reactive lymph nodes 12, and tuberculosis 1. Final diagnosis was established by C-TBNA in 14 (11 malignancy, 3 sarcoidosis; yield 41.1%), mediastinoscopy in 14, transthoracic needle aspiration in 3, peripheral lymph node biopsies in 2 and by endobronchial biopsy in 1. Nodal size had an impact on outcome (*P* = 0.000) while location did not (*P* = 0.33). C-TBNA was positive in 11/20 when malignancy was suspected (yield 55%), while 3/14 when benign diagnosis was suspected (yield 21.4%) (*P* = 0.05). Sensitivity, specificity, PPV, NPV, and diagnostic accuracy were 66.6%, 100%, 100%, 65%, and 79.4%, respectively. There were no complications or scope damage.

**CONCLUSION:**

Conventional-TBNA can be learned by the books and by practicing on inanimate models without formal training and results similar to those published in the literature could be achieved.

Interventional pulmonology (IP) is an evolving field within pulmonary medicine. It focuses on providing procedural services to patients with central airway disorders and pleural diseases. It requires additional training and expertise beyond that is acquired in a standard pulmonary medicine training program.[1] IP encompasses a large number of diagnostic and therapeutic procedures and the list is growing each day. Guidelines and consensus statements have been published to suggest a minimum number of procedures required to demonstrate a level of competence in the field.[2–3] IP fellowship programs offer scope for necessary skills and knowledge that a trainee should acquire within the field. These dedicated IP programs involve 1 year of additional training following standard pulmonary fellowship training.[4]

Unfortunately such IP programs are few and far between. To our knowledge, such training is available only in highly developed countries. Besides none of these programs have been systematically approved by the respective educational organization and there is lack of standardization of the curriculum. For the physicians practicing outside these countries such training is difficult to obtain; especially for the ones who are already in the clinical practice. Thus IP faces a challenge in dissemination of its art and science. Whilst Lung Cancer attains a pandemic proportion, practice of IP is in demand all over the world. Throughout the world, there are only a handful of interventional pulmonologists who have acquired their training through a formal fellowship program. The majority of pulmonologists gather their skills and experience via secondary means of education [Table 1].[1] Once again, it also needs to be noted that the majority of these secondary means are not accessible to most, especially those practicing the third world countries.

**Table 1:** Secondary means of interventional pulmonology training

Books, atlases and videos
On-line courses
Simulators
Postgraduate courses (1-3 days) with hands on session
Inanimate objects
Animal models
Simulators
Hands-on training session organized by the manufacturer of the equipment
Preceptorship
Mini-Fellowship (1-3 months)
Parent organization
Other organization

Additionally, the desire for training for a specific IP procedure is dictated by the regional epidemiology, local economics, and the interest of the pulmonologist. Thus training acquired during the formal IP training programs may be too vast and too time consuming for the most. In real practice, the majority of interested physicians gather the desired knowledge and skills by reviewing the literature, watching videos, and/or practicing on inanimate objects; one procedure at a time. However, the effectiveness of this practical approach has not yet been proven.

Conventional transbronchial needle aspiration (C-TBNA) has been proven to be a safe, minimally invasive, and cost-effective technique in establishing diagnosis of mediastinal pathologies.[5–7] Despite its established advantages, it still remains as an underutilized and underemphasized in the fellowship training programs. The limited acceptance of C-TBNA is presumed to be due to need for in-depth training and typical slow learning curve.[8] This may have lead to the belief that C-TBNA is not particularly worth for such a pursuit. In recent years, introduction of endobronchial ultrasound (EBUS) as well as esophageal ultrasound (EUS) in staging of lung cancer has further reduced interest in the training of C-TBNA; while availability of the instrument remains elusive in the developing countries.[9]

We believe that the procedure of C-TBNA is simple enough that it could be learned outside the IP fellowship program relying on the secondary measures. At our institution, we have no provision to formally acquire training in any aspect of IP. We acquired knowledge on the utility and technique of C-TBNA by the books and proceeded to perform it on our patient population. Our study is to prove the success of C-TBNA learned using the secondary measures.

## Methods

Baskent University School of Medicine is a tertiary care facility. There are 5 staff physicians and 10 fellows in the pulmonary diseases department. Approximately, 600/year conventional diagnostic bronchoscopies are performed at our institution. We do not perform any of the therapeutic procedures or advanced diagnostics, mainly due to the lack of required training in our country. We gathered working knowledge of C-TBNA by reviewing the literature,[10–14] watching video tapes (ConMed Corporation, Mentor, Ohio, USA, 2005 WANG^−^ Transbronchial Aspiration Needle, A Procedural Overview of Transbronchial Needle Aspiration), and by participating on inanimate model for TBNA (BARD Corporation, Boston, MA, USA as well as “Zavala Lung Model”). A book, “Flexible Bronchoscopy—2^nd^ edition (Editors: Ko-Pen Wang, Atul C. Mehta, J Frnacis Turner Jr, Blackwell Publishing, Cambridge, MA, USA, 2004) was used as a reference guide whenever needed. E.K. also participated at a hands-on training course organized by an international society in 2009. We have no access to simulators for bronchoscopy training. After improving skills and feeling confident about performing C-TBNA on the lung model, we started performing the actual procedure in December 2009, according to the description by Wang *et al*.[15] Since then the procedure was considered on consecutive patients presenting with mediastinal lymphadenopathy (MLA) on chest computed tomography (CT). All patients in whom the bronchoscopy was indicated for either diagnostic or staging purposes were included in the study. Patients in whom the procedure was contraindicated were excluded from the study. The study was carried out and the data were gathered in a prospective fashion. Chest CT was reviewed by both, the pulmonologist and the radiologist in detail to gauge the size and identify the location of the lymph nodes. The location was described according to Mountain’s classification.[16] Nodes were considered enlarged if its diameter in short axis was more than 10 mm.

The 21 and/or 19 gauge Smooth Shot Needles (Olympus^®^, Japan) were used at the discretion of the bronchoscopist. Latter gauge needle was mainly chosen if a benign diagnosis was suspected. Except for the gauge, there is no difference in the design or the insertion of both the needles. Once the 21 gauge needle was inserted to its fullest length, the catheter was agitated while applying suction at the proximal end using a 50 cc syringe to obtain loose cells for cytological examination. On the contrary, following its insertion the 19 gauge needed was moved back and forth by 2–3 mms through the tracheobronchial wall to obtain a core of specimen for histological examination. Tissue specimens were prepared according to the description by Wang *et al*.[15]

C-TBNA was routinely performed on all N2 and N3 lesions (if present) for staging of suspected bronchogenic carcinoma, and at N1 location for the purpose of making diagnosis. Rapid-on-site cytology examination (ROSE) was not available.[17] In cases where the lymph nodes (LN) from more than one location were sampled, the one with the largest size was taken into consideration for the calculations.

C-TBNA results were categorized into the following groups: malignant, non-malignant, or non-diagnostic. Diagnosis of malignancy was established based on cytology and/or histology findings. When tissue representative of a benign diagnosis (i.e.: Sarcoidosis) was present, the results were considered “confirmatory” for non-malignant LN. Both, malignant as well as benign diagnoses, were considered “true positive” if they matched our clinical suspicion, else further diagnostic steps were considered to rule out “false positive” results. The results were considered nondiagnostic if no material was obtained (Dry Tap) or if the procured material was not representative of any of the above two groups. In cases where the C-TBNA was either nondiagnostic or showed only normal lymphocytes, the final diagnosis was established by mediastinoscopy, transthoracic needle aspiration, and peripheral lymph node and/or endobronchial biopsies. Aspirates with normal lymphocytes were considered “true negative” if no definite diagnosis was established by any of the above methods.

Diagnostic yield, sensitivity, specificity, positive predicted value (PPV), negative predicted value (NPV), and diagnostic efficacy were defined as follows:

Diagnostic yield: Number of True Positives (TP)/Total number of procedures

Sensitivity: TP/TP + number of false negatives (FN)

Specificity: Number of true negatives (TN) / TN + Number of false positives (FP)

PPV= TP/TP + FP

NPV= TN/TN + FN

Diagnostic efficacy (accuracy): TP + TN/total patients × 100

(TP: Abnormal LN correctly diagnosed as abnormal (malignant as well as benign); FP: Normal LN incorrectly identified as abnormal; TN: Normal LN correctly identified as normal; FN: Abnormal LN incorrectly identified as normal)

All of the above values were expressed as the median and range for continuous variables. The influence of size and anatomical location of the lymph node on the outcome of C-TBNA was analyzed using χ^2^-test. All statistical tests were two-sided and *P* < 0.05 was considered statistically significant. All data were analyzed with a statistical software package (SPSS, version 11.5 for windows; SPSS inc; Chicago IL).

The study protocol was approved by the Ethics Committee, and all the patients signed an informed consent before the procedure.

## Results

Thirty-four patients (M:F = 23:11; 68%:32%) with mean age of 54.9 ± 11.8 (27-76) years underwent C-TBNA using either 21 g or 19 g (or both) SmoothShot Olympus^®^ needles for MLA. Twenty-two patients had LNs larger than 20 mms (mean: 33.0 ± 8.4 mm s) and the remainder between 10 and 20 mm (mean: 17.3 ± 2.1 mms). Locations of the target LNs were: 14 right paratracheal, 11 subcarinal, 7 right hilar, and 2 left hilar. Suspected diagnoses were: malignancy in 20 (lung cancer 18, lymphoma 1, and metastatic pancreatic cancer 1) and non-malignant condition in 14, [tuberculosis (TB) 8, sarcoidosis 6]. Final diagnoses were: lung cancer in 15, sarcoidosis in 4, reactive LNs in 12, TB, metastatic pancreas cancer and lymphoma, one of each. Final diagnosis was established by C-TBNA in 14 (diagnostic yield 41.1%), by mediastinoscopy in 14, by transthoracic needle aspiration in 3, by peripheral LN biopsies in 2, and by endobronchial biopsy in 1 patient. Satisfactory C-TBNA specimens were obtained from all aspirates except one.

**Figure 1:**
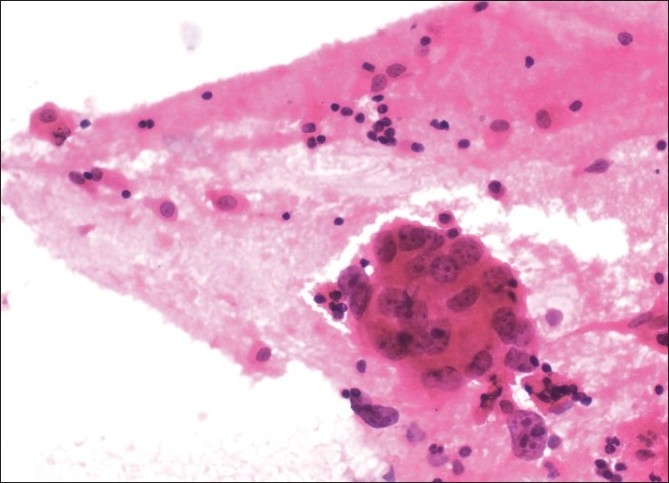
Adenocarcinomatous cells in the three-dimensional papillary group with vesicular nuclei, prominent nucleoli, and large amount of cytoplasm; Papanicolau stain, ×200

**Figure 2:**
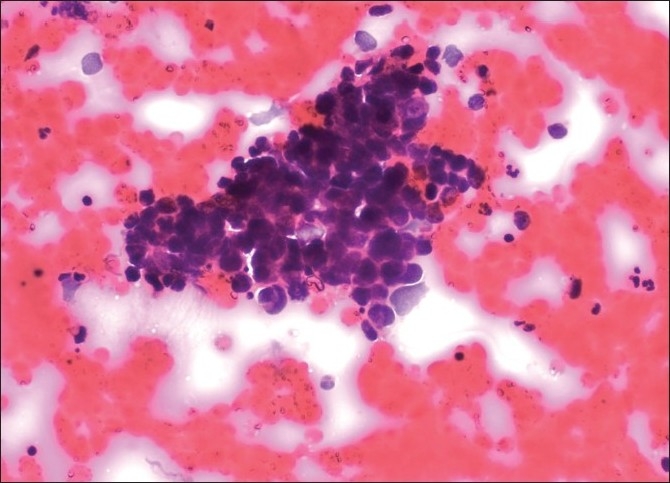
Small cell carcinoma: Hyperchromatic group of cells showing apoptosis, high nucleo-cytoplasmic ratio, and nuclear molding. Neoplastic cells do not show nucleoli; Papanicolau stain, ×200

**Figure 3:**
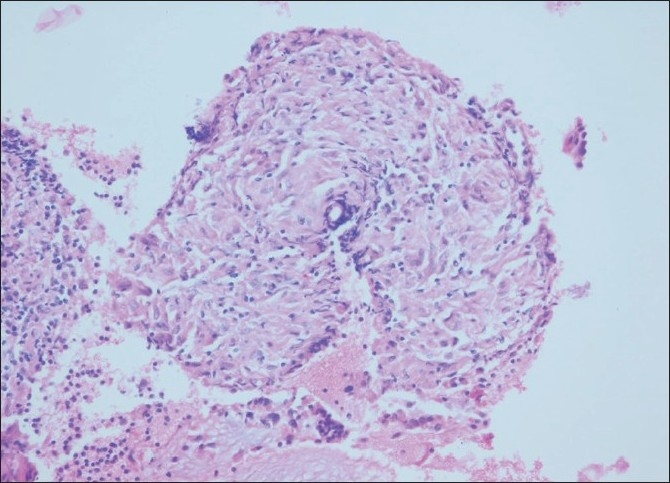
A large granuloma consisting of epitelioid histiocytes, few multinucleated giant cells, and lymphocytes; H and E, ×200

**Figure 4:**
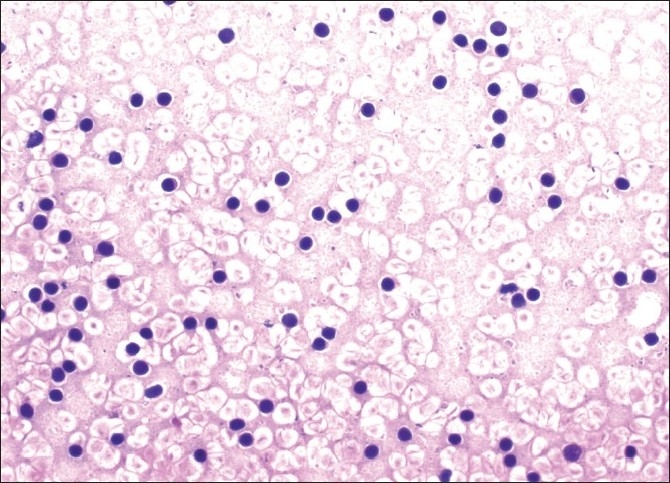
Benign reactive lymphocytes; H and E, ×100

C-TBNA revealed definitive diagnosis in 14 patients (10 Lung Ca, 1 metastatic pancreas cancer, 3 sarcoidosis) [Figures 1–3]; 10 aspirate revealed normal lymphocytes [Figure 4] while 9 specimens were of limited cellularity for a specific comment. There was one dry tap. The lymph node size had an impact on the outcome of TBNA (*P* = 0.000) (diagnostic yield in <20 mm LNs: 0%, diagnostic yield in >20 mm LNs: 63.6%), while location did not (*P* = 0.33) [Table 2]. C-TBNA was positive in 11/20 when malignant diagnosis was suspected (diagnostic yield 55%), while in cases with suspected nonmalignant diagnosis it was positive 3/14 (diagnostic yield 21.4%) and this difference was statistically significant (*P* = 0.05) [Table 3]. We did not encounter any positive results that did not match our clinical suspicion (false positive). C-TBNA had a higher diagnostic yield in malignancy (11/17) than in the non-malignant processes (3/17) (64.7% vs. 17.6%, *P* = 0.005). Sensitivity and the specificity of C-TBNA were 66.6% and 100%, respectively. The PPV was 100% and the NPV was 65%. The overall accuracy of the procedure was 79.4% [Table 4]. We did not experience any specific difficulty in using either 21 or 19 gauge needles. We encountered neither any complications nor damage to the bronchoscope.

**Table 2:** The outcome of TBNA according to the lymph nodes location and the size of the lymph nodes

	TBNA diagnosis		P
	(+)	(−)	
Location			
Paratracheal	6	8	0.33
Subcarinal	6	5	
Hilar	2	7	
Size			
<20 mm	0	12	0.000*
>20 mm	14	8	

* *P*< 0.05: statistically significant; TBNA: Transbronchial needle aspiration

**Table 3:** The diagnostic yield of TBNA in malignant and benign diseases

Suspected diagnosis	N	TP TBNA	Diagnostic yield (%)
Malignant *	20	11	55
Benign**	14	3	21.4
Overall yield	34	14	41.1

TP: True positive;

* Malignant: Lung Ca, lymphoma, metastatic pancreas cancer;

** Benign: Sarcoidosis, tuberculosis; TBNA: Transbronchial needle aspiration

**Table 4:** The sensitivity, specificity, PPV, NPV and the diagnostic accuracy

Suspected diagnosis	Specificity%	Sensitivity%	PPV	NPV	Accuracy%
Malignant	100	68.7	100	44.4	44.1
Benign	100	60	100	81.8	35.2
Over all	100	66.6	100	65	79.4

PPV: Positive predicted value, NPV: Negative predicted value

## Discussion

C-TBNA provides an opportunity to diagnose mediastinal lesions and stage lung cancer in a minimally invasive fashion.[2,3] Despite such advantages, TBNA is underutilized,[8] most likely due to lack of appropriate training.[18] We adopted the procedure of C-TBNA at our institution strictly from the secondary means of training.

The yield of C-TBNA in the diagnosis and staging of lung cancer has been reported between 20% and 80% in the literature.[19–24] Single meta-analysis found sensitivity and specificity of C-TBNA for the diagnosis of nonsmall cell lung cancer to be 39% and 99%, respectively.[5] These values were dependent on the prevalence of lung cancer within the population. In our population, the sensitivity and the diagnostic yield of C-TBNA for lung cancer have been found to be between 58-70% and 60-100%, respectively [Table 5].[22,23,25–28] In our study the sensitivity of C-TBNA for lung cancer was 66.6% which matches with the literature. Our diagnostic yield is lower than that is reported in the literature, yet it is still within the range and could still represent our learning curve.

**Table 5:** The diagnostic yield of TBNA for mediastinal lymphadenopathy in different studies from Turkey

Study	n	Overall DY %	DY lung Ca %	DY sarcoidosis %	DY TB %	Sensitivity %
Bilaceroglu[47]	84	78	-	-	75	83 (TB)
Bilaceroglu[30]	74	-	-	61% stage1 42% stage2	-	-
Bilaceroglu[23]	138	-	78	-	-	70 (Lung ca)
Bayram[25]	55	-	60	-	-	58 (Lung ca)
Çetinkaya[22]	60	50	100	76	65	-
Çetinkaya[24]	28	87	-	87.5	100	-
Küpeli present study	34	41.1	66.6	75	0	66.6 (Lung Ca and overall)

DY: Diagnostic yield, Lung Ca: Lung cancer, TB: Tuberculosis, TBNA: Transbronchial needle aspiration

In the literature, the diagnostic yield of C-TBNA for sarcoidosis varies between 50% and 72%.[22,24,29–31] Few reports published from Turkey revealed this diagnostic yield between 42% and 87.5%.[22,24,30] Our diagnostic yield for sarcoidosis was 75% which was within the range with the other studies from our country as well as in the literature yet, the number of patients studied in our group was too small.

Although C-TBNA is a simple technique for the diagnosis of MLA, it continues to be underutilized.[32] Of the several reasons provided for such practice, the perceived impediment is availability of proper training. This is based on the fact that a survey found that most pulmonary fellows thought they had been adequately trained in FB, but only 72.9% had received any instructions on C-TBNA.[33]

Self-learning experience and the learning curve of C-TBNA in the hands of physicians using secondary means of training has also been executed in various studies in the world’s literature.[19,34–41] Hermens *et al*. reported their experience with C-TBNA with self-training of 5 days by five different pulmonologists and attained a diagnostic yield of 77%.[39] Boonsarngsuk *et al*. reported the diagnostic yield of 84.6% after learning the procedure from videotapes and training on a lung model.[40] In 1995, Haponik *et al*. revealed that following 3 years of practicing, the C-TBNA yield increased 2 folds while the incidence of unsatisfactory specimens decreased five folds.[36] Similarly, in 1997, De Castro *et al*. reported that after 24 months of training period the sensitivity of C-TBNA improved from 32% to 78%.[37] In order to achieve acceptable results, it was estimated that approximately 50 procedures were required to attain proficiency. In 2004, Hsu *et al*. reported a high diagnostic accuracy (75.9%) and continued improvement in sensitivity of C-TBNA during a 4 year of learning period.[19] In 2006, Raveglia *et al*. attained a diagnostic yield of 61% over an 18-month period of training.[38] CT fluoroscopy-guided TBNA and ROSE have been shown to significantly increase in the diagnostic yield of C-TBNA.[17,42,43] Unfortunately, these services are not available at our institution.

In the present study, LN size was found to be significantly associated with C-TBNA diagnostic yield similar to other studies.[44–46] On the other hand, we could not demonstrate any significant effect of the LN location on C-TBNA yield yet that could be due to the small size of our study.[17,42,44,45] In the benign diseases, the diagnostic yield of TBNA was lower when compared with malignant diseases consistent with the literature,[44,45] despite the use of the histology needle.

We accept that there are several weaknesses in our study. First, that it is a preliminary study and that we are still in our learning curve. We were also not able to make the diagnosis if the LNs were less than 20 mm in size. Second, we have a small number of patients. Besides we did not use ROSE which has been shown to significantly increase the diagnostic yield of TBNA and reduce the number of dry taps.[17]

It was a coincidence that the majority of our patients had LNs larger than 20 mm in size and we failed to make the diagnosis from the smaller ones. However, we were able to retrieve reactive lymphocytes from these smaller LNs, indicating that we were successful in reaching our targets. Yet, we are confident that our yield will continue to improve with experience. The objective of our study was to perform C-TBNA primarily to establish diagnosis and not for staging. Thus, if TBNA results were positive (diagnostic) and matched our clinical suspicion we did not pursue further confirmation. In all patients TBNA results paralleled clinical suspicion and in our judgment there were no false positive results; hence our specificity was 100%. Once again, based on our primary objective of establishing the diagnosis, we calculated our results based on the number of the patients than the number of lymph nodes aspirated. On the contrary the strengths of study are its prospective nature, consecutive recruitment, and establishment of final diagnosis in all patients. Besides we did not experience any complications or scope trauma.

In conclusion, C-TBNA is a safe, simple, and a reliable technique. It remains underutilized due to lack of required formal training. Our study proves that the procedure can be successfully learned without formal training that is offered in IP programs. In other words, TBNA can be learned “by the books”; post-graduate courses, workshops, or hands-on courses can certainly add more to the initial exposure to the procedure. Even in the era of EBUS as well as esophageal ultrasound (EUS), acquiring skills to perform C-TBNA is essential as availability of the instrument and accessories remains elusive in the developing countries.[9] Besides, as seen in our scenario obtaining training and maintaining skills at EBUS and EUS is much more difficult than with C-TBNA.

Availability of formal IP training is scarce, while the need for demand for the services is disproportionately high. Centers of excellences can certainly offer comprehensive services and required training to highly select, exceptional individuals. Invasive procedures such as rigid bronchoscopy or the silicon stent placement may continue to remain within the domain of such centers while the community physicians can learn selected IP techniques by the books. Manufactures of the bronchoscopy equipment continue to provide hands-on courses using experts from the centers of excellences. Competence gained through such means should be thus accepted and respected to further the welfare of patients in the third world countries. Selection of the procedures learned by the books depends upon the regional epidemiology, economics, and physician’s interest.
